# Facial skin metastasis due to small-cell lung cancer: a case report

**DOI:** 10.1186/1752-1947-3-32

**Published:** 2009-01-29

**Authors:** Nikolaos Barbetakis, Georgios Samanidis, Dimitrios Paliouras, Elpida Samanidou, Zoi Tzimorota, Christos Asteriou, Persefoni Xirou, Christodoulos Tsilikas

**Affiliations:** 1 Thoracic Surgery Department, Theagenio Cancer Hospital, A. Simeonidi, Thessaloniki, 54007, Greece; 2 General Surgery Department, Theagenio Cancer Hospital, A. Simeonidi, Thessaloniki, 54007, Greece; 3 Plastic Surgery Department, Theagenio Cancer Hospital, A. Simeonidi, Thessaloniki, 54007, Greece; 4 Pathology Department, Theagenio Cancer Hospital, A. Simeonidi, Thessaloniki, 54007, Greece

## Abstract

**Introduction:**

Cutaneous metastases in the facial region occur in less than 0.5% of patients with metastatic cancer. They are an important finding and are not often the first sign leading to diagnosis.

**Case presentation:**

We describe the case of a 64-year-old male patient who presented with dyspnea, pleuritic pain, loss of weight and a nodule on his left cheek. A chest X-ray revealed a left upper lobe mass with mediastinal lymphadenopathy. Excision biopsy of the facial nodule revealed small-cell lung carcinoma. Palliative chemo-radiotherapy was administered and the patient survived for 12 months.

**Conclusion:**

A high index of suspicion is necessary for the early detection of facial cutaneous metastases. Appropriate treatment may prolong patient survival.

## Introduction

Cutaneous metastases in the facial region occur in less than 0.5% of patients with metastatic cancer, and they usually originate from malignant melanoma [1]. Various types of pulmonary cancer lead to cutaneous metastases in 1.5 to 2.6% of cases [2]. In this report, we describe an unusual case of small-cell lung cancer metastasizing to his face at the time of initial diagnosis.

## Case presentation

A 64-year-old man, a heavy smoker, was referred to our department with a short history of dyspnea, pleuritic pain and loss of weight, as well as a painful nodule on his left cheek which was noticed almost simultaneously with the principal symptoms. His general condition was good, although he suffered from coronary artery disease and diabetes mellitus type II. A chest X-ray revealed a left upper lobe mass with mediastinal lymphadenopathy without pleural effusion. Bronchoscopy revealed no evidence of malignancy, and bronchial biopsy and washings also proved negative for malignant cells. In order to perform pre-operative staging of the tumour, the patient underwent computed tomography (CT) scans of brain and abdomen, and a bone scan. All had normal results.

Cutaneous examination at the time showed a 1.5 cm painful nodule on the patient's left cheek. The adjacent skin had inflammatory signs. Physical examination showed nothing abnormal, with no palpable lymph nodes or nodules.

**Figure 1:**
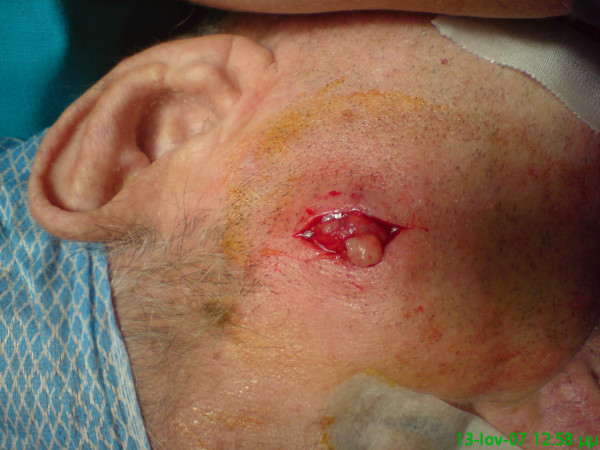
**A 1.5 cm nodule was excised**.

The patient underwent excision biopsy of the facial lesion (Figure 1). Subsequent histological sections showed infiltration by small-cell lung carcinoma (SCLC). A CT-guided biopsy of the lung tumour confirmed the presence of a SCLC and chemo-radiotherapy was initiated. The patient survived for 12 months. He died due to respiratory insufficiency with additional bone and brain metastases.

## Discussion

SCLC results from bronchial epithelial cells, which are relatives of Kultchitsky cells, a type of intestinal epithelial cell. SCLC is fatal and most patients die within one year of presentation. When untreated, patients survive only for one to three months after diagnosis. Survival is short even when patients are treated, due to the aggressive biological behaviour of this type of tumour. The mainstay of treatment is chemotherapy combined with radiotherapy with a mean survival period of 8 to 15 months. The disease most frequently metastasizes to the central nervous system, bone marrow and suprarenal glands. SCLC may be accompanied by paraneoplastic syndromes, superior-vena-cava syndromes, compressions to the spinal cord and, very rarely, skin metastases [3]. According to the literature, the various types of lung cancer lead to cutaneous metastases in 1.5% to 2.6% of cases. Furthermore, in a review of 4020 cases of cutaneous metastases from systemic cancers, only 19 were pulmonary and only two of those were from small-cell carcinoma, the latter tending to metastasize at the back [4]. In a recent original paper on cutaneous metastases, lung cancer is the second most common cause (as many as 8 out of 32 reported cases), and the upper trunk and the abdomen were the most frequent sites, followed by the head and neck [5].

**Figure 2:**
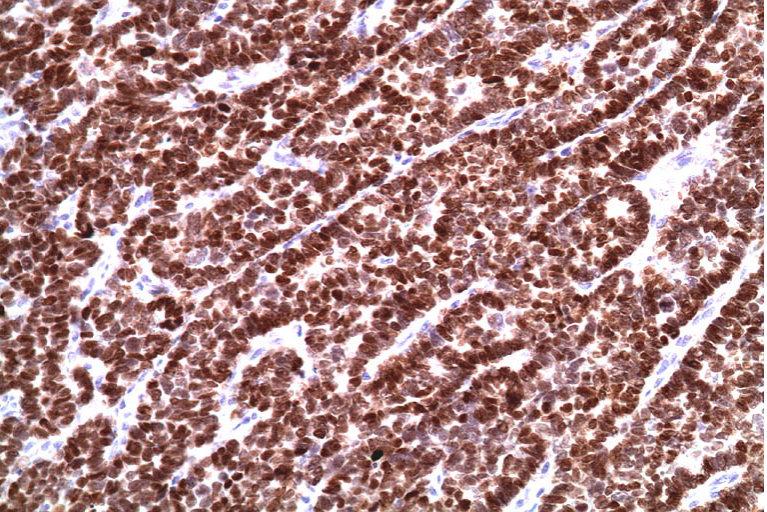
**Immunohistochemical stain with thyroid transcription factor 1 was positive**.

**Figure 3:**
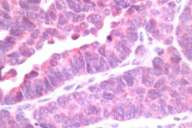
**The neuro-endocrine markers of neuron-specific enolase were positive**.

**Figure 4:**
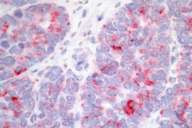
**The neuro-endocrine markers of chromogranin were positive**.

Cutaneous metastases as a first sign of internal malignancy occur infrequently. Clinically, they manifest as nodules, ulceration, cellulitis-like lesions, bullae or fibrotic processes [6]. The differential diagnoses considered clinically, along with a metastatic carcinoma of the lung, were squamous-cell carcinoma, basal-cell carcinoma, amelanotic melanoma, carcinoid tumour, Merkel-cell carcinoma, neuro-endocrine carcinoma, malignant fibrous histiocytoma, atypical fibroxanthoma and dermatofibrosarcoma protuberans. In our case, cytokeratin 20 was negative, ruling out Merkel-cell carcinoma. Immunohistochemical staining with thyroid transcription factor (TTF-1) was positive, confirming that it was primary in the lung (Figure 2). The neuro-endocrine markers of neuron-specific enolase (NSE) and chromogranin were positive (Figures 3 and 4). The combination of TTF-1, NSE and chromogranin-positivity led to the diagnosis of SCLC. Carcinoid tumours are typically TTF-1-negative and show positivity with NSE and chromogranin. The histological pattern ruled out the remaining differential diagnoses.

Generally, cutaneous metastases are early indicators of metastatic disease. Diagnosis may be delayed by several months, unless the skin lesion grows rapidly or other sites such as the lung or liver are affected by the tumour's spread [7]. Early recognition of tumour relapse from a suspicious skin lesion may lead to initiation of treatment before widespread metastases occur [6]. In our case, the facial metastasis was found simultaneously with the primary lung tumour, facilitating diagnosis.

## Conclusion

Despite the fact that skin metastasis has poor prognosis, a high index of suspicion is necessary for its early detection. The aim is to start treatment as soon as possible before widespread visceral metastases occur. Therefore, close inspection of new skin lesions in patients with a history of malignancy is imperative, and diagnostic biopsy is always essential.

## Abbreviations

CT: computed tomography; NSE: neuron-specific enolase; SCLC: small-cell lung carcinoma; TTF-1: thyroid transcription factor;

## Consent

Written informed consent was obtained from the patient's family for publication of this case report and accompanying images. A copy of the written consent is available for review by the Editor-in-Chief of this journal.

## Competing interests

The authors declare that they have no competing interests.

## Authors' contributions

NB, GS, DP, ES, ZT and CA took part in the care of the patient and contributed equally to the medical literature search. PX was responsible for the pathology report. CT participated in the care of the patient and supervised this report. All authors approved the final manuscript.
